# Spontaneous Escherichia coli Empyema Thoracis: An Unusual Occurrence in a Non-cirrhotic, Immunocompetent Individual

**DOI:** 10.7759/cureus.26618

**Published:** 2022-07-06

**Authors:** Riffat Sabir, Muhammad Umar, Mehak Ali

**Affiliations:** 1 Internal Medicine, Baystate Medical Center, Springfield, USA; 2 Hospital Medicine, Baystate Medical Center, Springfield, USA

**Keywords:** complex medical history, high aspiration risk, oropharyngeal dysphagia, pulmonary thromboembolism, non-cirrhotic, escherichia coli empyema, spontaneous bacterial empyema

## Abstract

Spontaneous bacterial empyema is a spontaneous infection of the pleural cavity in the absence of pneumonia, typically seen in patients with liver cirrhosis and preexisting hepatic hydrothorax. Empyema thoracis caused by *Escherichia coli *(*E. coli*) is a rare clinical finding and, in most cases, a consequence of *E. coli* pneumonia. Spontaneous bacterial empyema secondary to *E. coli *in a non-cirrhotic individual is an unusual association, rarely described in the literature. To the best of our knowledge, this is the first case of spontaneous bacterial *E. coli* empyema thoracis in a non-cirrhotic, immunocompetent individual with a complex medical history including pulmonary thromboembolism, oropharyngeal dysphagia, and a high aspiration risk of oropharyngeal secretions.

## Introduction

Empyema thoracis, defined as a collection of pus in the pleural space, was first recognized by Hippocrates more than two millennia ago [1]. Despite being a historical disease, much still needs to be explored about the pathophysiology and microbiology of empyema. Thoracic empyema is commonly attributed to pneumonia, thoracic trauma, iatrogenic procedures including thoracentesis and cardiac surgery, bronchopleural fistula, esophageal rupture, and extension of infection from the abdomen and mediastinum, amongst many others [2]. Spontaneous pleural space infection without the aforementioned predisposing conditions is rare, primarily seen in patients with cirrhosis, and known as spontaneous bacterial empyema (SBEM).

The microbiologic spectrum of thoracic empyema can be broad, depending primarily on the acquisition route, i.e., parapneumonic or non-parapneumonic. Parapneumonic empyema is commonly caused by *Streptococcus pneumonia*, *Staphylococcus aureus*, and anaerobes [3]. Non-parapneumonic empyema, on the other hand, can be caused by a diverse category of organisms and varies with the underlying source of infection. *Escherichia coli* (*E. coli*) and other gram-negative Enterobacteriaceae collectively account for 8 to 10% of cases. Regardless of the cause and source of infection, empyema carries a high mortality and morbidity ranging from 20 to 30% [4]. Hence, early diagnosis and management become critical in improving clinical outcomes and survival.

We present a case of unilateral SBEM and bacteremia caused by *E. coli* in a non-cirrhotic, immunocompetent individual with a complex medical history including pulmonary thromboembolism, oropharyngeal dysphagia, and a high aspiration risk of oropharyngeal secretions.

## Case presentation

A 100-year-old male presented to the emergency department (ED) with a two-day history of generalized weakness and worsening exertional dyspnea. His medical history was significant for advanced dementia, severe aortic stenosis status post-transcatheter aortic valve replacement and permanent pacemaker implantation, New York Heart Association (NYHA) III heart failure with reduced ejection fraction (EF) 35%, paroxysmal atrial fibrillation; not on long-term anticoagulation, infrarenal abdominal aortic aneurysm, colon cancer in remission status post right hemicolectomy, remote history of hemorrhagic stroke, partial dependency for all activities of daily living, and instrumental activities of daily living.

On admission, his vitals were as follows: blood pressure 111/77 mmHg, pulse 128/min, temperature 101.8°F, respiratory rate 28/min, saturating 98% on 3 L/min of supplemental oxygen. He was alert, awake, and inconsistently responding to questions, which was his baseline as per family. The respiratory examination was remarkable for bilateral expiratory wheezing with decreased breath sounds over the left lung base. Cardiovascular examination revealed an irregular rhythm and a trace lower extremity pitting edema without jugular venous distention. The abdomen was soft, non-distended, with no shifting dullness. The remainder of the physical examination was unremarkable.

**Figure 1:**
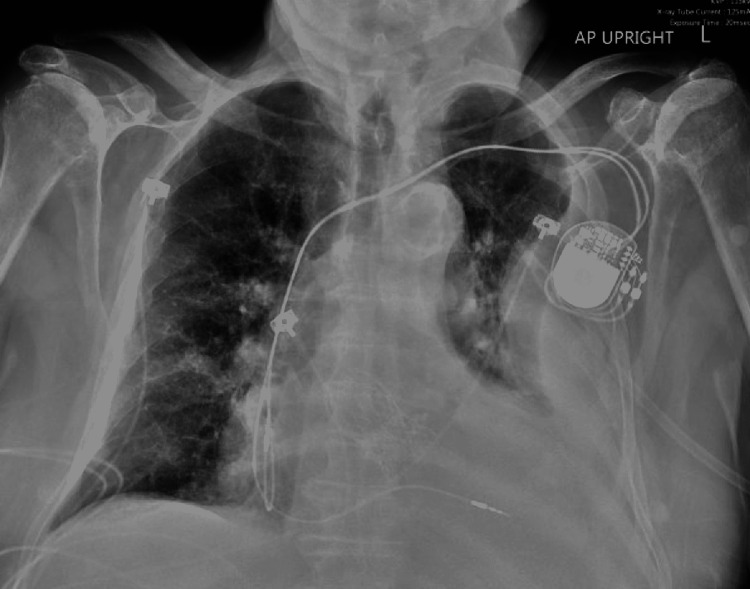
Chest X-ray anteroposterior view demonstrating left pleural effusion

Initial laboratory workup was notable for a white blood count (WBC) of 15.7 k/mm^3^ (normal range 4-11 k/mm^3^) with 83% neutrophils, lactate 3.8 mmol/L (normal range 0.5-2.2 mmol/L), with negative urinalysis and respiratory viral pathogen panel. Liver function tests showed aspartate transaminase 43 U/L (normal range 0-33 U/L), alanine transaminase 32 U/L (normal range 0-41 U/L), alkaline phosphatase 151 U/L (normal range 40-129 U/L), total bilirubin 2.1 mg/dL (normal range 0-1.2 mg/dL) with a direct fraction of 1.3 mg/dL. Right upper quadrant abdominal ultrasound was negative for hepatobiliary pathology. X-ray imaging of the chest showed a moderate-sized left-sided pleural effusion (Figure 1).

**Figure 2:**
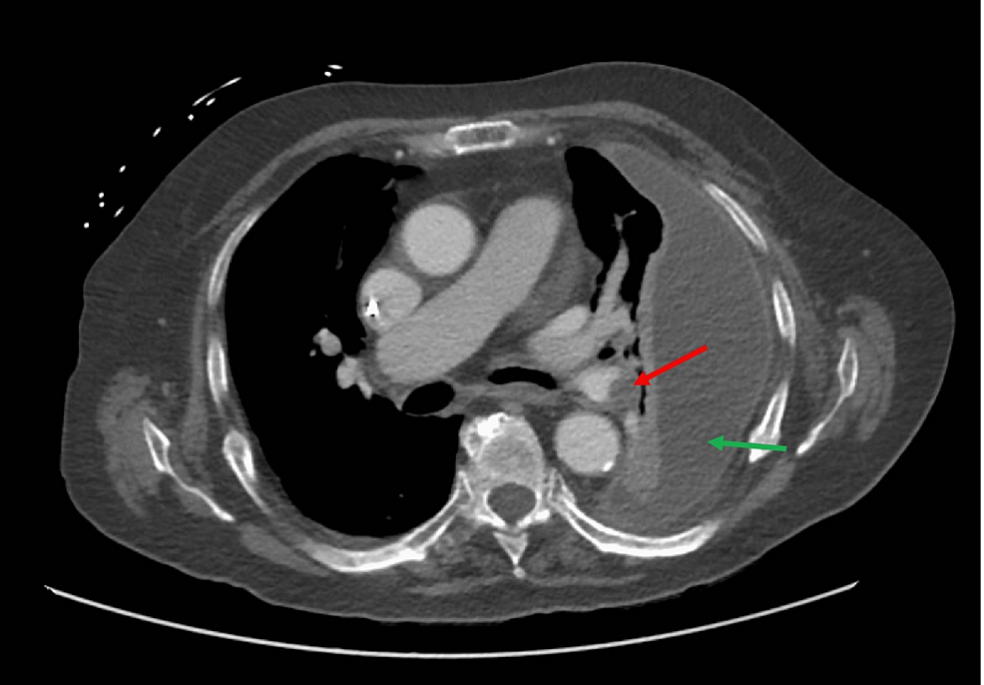
Computed tomography of the chest demonstrating pulmonary thromboembolism in the left main pulmonary artery (red arrow) and large loculated left-sided pleural effusion (green arrow)

**Figure 3:**
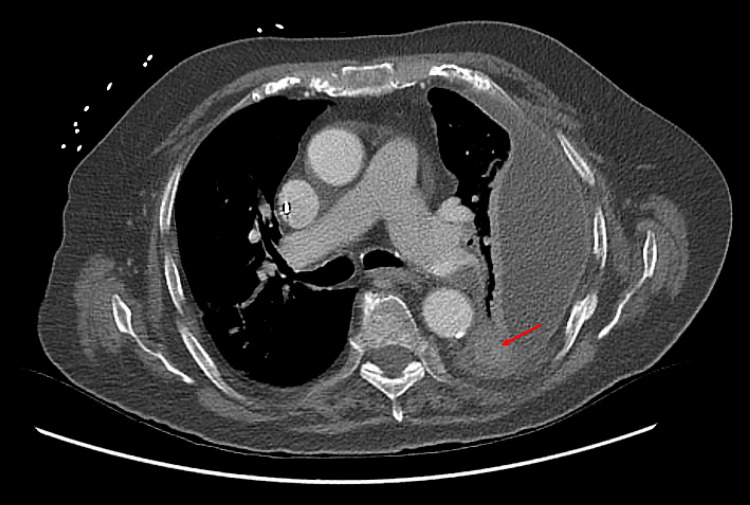
Computed tomography of the chest demonstrating calcifications in the left lower lobe of the lung, suggestive of microaspirations (red arrow)

An initial diagnosis of sepsis likely secondary to community-acquired pneumonia was made, and he was started on intravenous ceftriaxone and doxycycline. A day later, *E. coli* was isolated in the blood cultures drawn at admission. To elucidate the source of *E. coli* bacteremia further, contrast-enhanced computed tomography (CT) scanning of the chest, abdomen, and pelvis was obtained. The imaging demonstrated a large encapsulated left pleural effusion without lung consolidation, pulmonary embolism (PE) involving the left main pulmonary artery extending into the lingular and left lower lobe branches, and elevated right-to-left ventricular (RV/LV) diameter ratio >1, suggesting right heart strain or chronic sequelae of pulmonary hypertension. (Figure 2). It also showed calcification within the left lower lobe, consistent with prior aspiration events. (Figure 3). No ascites or cirrhotic morphology of the liver was noted on the CT of the abdomen.

The patient subsequently underwent a left-sided chest tube thoracostomy the same day, and 700 cc of purulent fluid was drained. Pleural fluid analysis showed the following: pH 7.19, glucose <2 mg/dL, lactate dehydrogenase (LDH) 3417 units/L, and WBC 152,260 cells/mm^3^ with 91% neutrophils (Table 1). According to Light’s criteria, the pleural fluid was consistent with an exudative effusion with a protein ratio > 0.5, LDH ratio > 0.6, and pleural fluid LDH level > two-thirds the upper limit of serum LDH. The pleural fluid also fulfilled the criteria for empyema given aspiration of grossly purulent material on thoracentesis, pH <7.2, glucose <40 mg/dL, and LDH >1000 IU/L. The culture of the pleural fluid later yielded *E. coli* as well. Ceftriaxone dose was increased to 2 g daily for bacteremia, and a heparin drip was initiated for anticoagulation. Intrapleural fibrinolytic therapy was deferred as the patient was on concurrent anticoagulant therapy and was deemed a poor candidate for surgical management of empyema.

**Table 1:** Pleural fluid analysis

Pleural Fluid Analysis		
Laboratory Test	Results	Reference Value
Color	Amber	Clear or yellow
Appearance	Cloudy	Clear
pH	7.19	7.60-7.64
Glucose	<2 mg/dl	Similar to plasma level
Protein	3.6 g/dL	1-2 g/dL
Pleural-serum protein ratio	0.58 (> 0.5)	N/A
LDH	3,417 units/L	<50% of plasma level
Pleural-serum LDH ratio	16 (> 0.6)	N/A
WBC	152,260 cells/mm^3^	1000-5000 cells/mm^3^
Differential %	Neutrophils 91%, lymphocytes 1%	Neutrophils 10%, lymphocytes 2-30%
Culture	Escherichia coli	Negative
Cytology	Negative	Negative

In addition, a transthoracic echocardiogram was completed, showing severely reduced right and left ventricular systolic function (EF could not be assessed given inadequate image quality) and borderline pulmonary hypertension with an estimated pulmonary artery systolic pressure of 40 mmHg (Figure 3 and Video 1).

**Figure 4:**
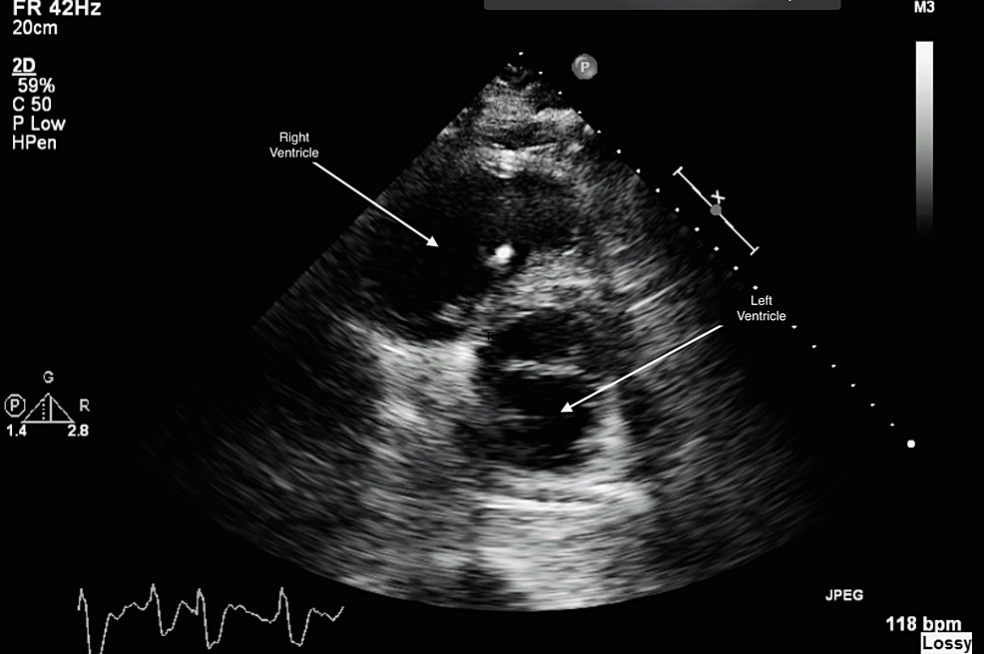
TTE parasternal short-axis view at mid-left ventricular cavity level demonstrating reduced biventricular function TTE: transthoracic echocardiography

**Video 1:** TTE parasternal long-axis view demonstrating severely reduced left ventricular ejection fraction TTE: transthoracic echocardiography

The patient’s swallowing assessment confirmed mild to moderate oropharyngeal dysphagia. His medical history of advanced dementia and stroke did place him at a higher risk of oropharyngeal dysphagia with a subsequent high aspiration risk of oropharyngeal secretions. Upon further review of the patient’s medical records, it was noted that the left-sided pleural effusion was also present on an X-ray chest obtained six weeks before this hospitalization. That X-ray was completed as a part of the patient’s radiologic trauma assessment in the ED after sustaining a mechanical fall at home. Since he was asymptomatic from a respiratory standpoint, no further workup was pursued at that time. There is a high likelihood that the left-sided pleural effusion developed in the setting of ipsilateral pulmonary thromboembolism, which then transformed into empyema due to direct pleural space infection from microaspiration events, as there was no clinical or radiological evidence of pneumonia.

Unfortunately, the patient developed delirium during the hospitalization, and his clinical condition continued to worsen despite targeted antibiotic therapy, chest tube drainage, and anticoagulation therapy. The comfort-directed approach was pursued per family wishes, and the patient was discharged home with hospice services on day eight of hospitalization. He later passed away peacefully at home.

## Discussion

SBEM is a rare entity, defined as a spontaneous pleural infection without underlying pneumonia. The diagnostic criteria include pleural fluid with a polymorphonuclear (PMN) cell count > 500 cells/mm^3^ or positive culture with PMN cell count > 250 cells/mm^3^ with the exclusion of pneumonia or a contiguous infection process on chest radiography [5]. SBEM is estimated to occur in 2.0% to 2.4% of cirrhotic patients without hydrothorax and 13% to 16% of cirrhotic patients with hepatic hydrothorax [6].

The pathogenesis of SBEM in cirrhotic patients remains unclear. Direct bacterial spread from the peritoneal cavity is likely the pathogenic mechanism for many; however, nearly 40% of the SBEM episodes are not related to spontaneous bacterial peritonitis, and SBEM may still occur in the absence of ascites. In those cases, transient bacteremia with resultant infection of the pleural space is attributed as the underlying mechanism [7]. In addition, patients with lower levels of pleural fluid complement C3, low ascitic fluid opsonic activity, and a higher Child-Pugh score are at a higher risk of developing SBEM than others [8].

The incidence of SBEM in non-cirrhotic individuals is scarce. Only a few cases of SBEM in patients without cirrhosis and ascites have been reported in the literature [9-11]. Chen et al. reported a case of *E. coli* and *Aeromonas hydrophila* empyema in a patient with nephrotic syndrome secondary to membranous nephropathy on steroids and cyclophosphamide [9]. Nguyen et al. reported a case of *Streptococcus pneumoniae* empyema and bacteremia in a patient with poorly controlled diabetes mellitus, alcoholism, congestive heart failure (CHF), and hemodialysis-dependent renal disease [10]. Lastly, Lourdusamy et al. reported a case of *E. coli* empyema in a patient with end-stage renal disease status post-renal transplant with subsequent chronic allograft rejection on immunosuppressive therapy [11]. In all these patients, a co-existent immunosuppressive state likely predisposed them to the development of SBEM.

SBEM is associated with high mortality; therefore, management should be prioritized and individualized. The recommended treatment for SBEM in cirrhotic individuals includes broad-spectrum antibiotics that should be tailored to the culture and sensitivity of the identified organism [5]. Chest tube thoracostomy in such patients can lead to life-threatening electrolyte imbalance and fluid and protein depletion and should generally be avoided. On the other hand, SBEM in non-cirrhotic individuals should be managed similarly to parapneumonic empyema with antibiotics, chest tube thoracostomy, intrapleural fibrinolytic therapy, and surgical management when indicated [12].

*E. coli* is a gut commensal and, therefore, often overlooked as a respiratory pathogen. Pulmonary infections from *E.coli* are rare, commonly associated with hematogenous dissemination from a primary source in the urinary and gastrointestinal tract, and less likely to aspiration of oropharyngeal secretions due to colonization [13]. Oropharyngeal colonization from *E. coli* is itself an uncommon occurrence. A multifactorial process, including prolonged supine position, aspiration of gastric contents, and altered gastric pH from proton inhibitors in patients with impaired local immunity and frequent exposure to the healthcare system, is attributed to *E. coli* oropharyngeal colonization [14]. Elderly patients with associated chronic illnesses, including diabetes mellitus, renal disease, and alcoholism, are at a higher risk of pulmonary infections from *E. coli *than the general population [15].

PE, a leading cause of undiagnosed exudative pleural effusions, has an estimated prevalence of 19-61% in patients with pleural effusion [16]. PE is also the fourth leading cause of pleural effusion in the United States after CHF, parapneumonic effusion, and malignancy [17]. The mechanism of pleural effusion in PE is an increase in the pulmonary capillary permeability and accumulation of interstitial fluid in the lungs secondary to the release of vascular endothelial growth factors and vasoactive cytokines. Multiple identifiable risk factors, including advanced age, obesity, previous venous thromboembolism, recent surgery, immobilization, trauma, active malignancy, active autoimmune disease, active inflammatory bowel disease, nephrotic syndrome, hormonal therapy, and hereditary and acquired thrombophilia, among many others, are implicated in the causation of PE. However, one-fifth of the patients are found to have unprovoked PE with no identifiable risk factors [18].

Our patient did not have clinical or radiological evidence of pneumonia, cirrhosis, or ascites. He had no history of the lymphoproliferative disorder, autoimmune disease, active malignancy, alcohol abuse, or other immunosuppressive conditions. He carried a diagnosis of colon cancer, for which he had undergone a right hemicolectomy in the remote past. His subsequent post-treatment surveillance colonoscopies and CT of the abdomen and pelvis were notably negative for the neoplastic process within the abdomen and pelvis; therefore, surveillance testing was concluded at the age of 84. During his current hospitalization, the family denied recent weight loss or change in bowel habits, and the CT abdomen and pelvis didn’t reveal a colonic mass, colonic wall thickening, luminal narrowing, intraabdominal and pelvic lymphadenopathy, or any other clinical or radiological findings to suspect colon cancer recurrence. This makes us believe he was an immunocompetent individual with a complex medical history. His history of advanced dementia and stroke predisposed him to oropharyngeal dysphagia with a resultant high risk of aspiration. The CT chest during current admission also demonstrated the presence of calcifications consistent with previous aspiration events within the left lower lobe, which was verified with the identification of mild to moderate oropharyngeal dysphagia on swallowing assessment. Therefore, he chronically suffered from silent microaspirations of oral secretions secondary to his swallowing dysfunction.

Our patient most likely developed spontaneous *E. coli* empyema by microaspiration of oropharyngeal secretions previously colonized by this organism, followed by direct microscopic bacterial invasion of the pleura and pleural space. The pleural space infection was possible in the presence of a preexisting left-sided pleural effusion, likely secondary to the ipsilateral PE. Any possible association of the pleural effusion with CHF, parapneumonic effusion, and malignancy was unlikely as there was a lack of clinical and radiological evidence to support either diagnosis. As outlined above, our initial working diagnosis did include left-sided community-acquired pneumonia with concurrent parapneumonic effusion; however, the CT chest didn’t reveal pulmonary consolidation. Therefore, ipsilateral PE is the likely explanation for left-sided pleural effusion. We suspect advanced age and baseline sedentary behavior as our patient’s identifiable risk factors for PE. He didn’t have a history of venous thromboembolism, and bilateral venous doppler completed during this hospitalization was negative for lower extremity deep venous thrombosis. He had been on anticoagulation in the past for paroxysmal atrial fibrillation, which was discontinued after the hemorrhagic stroke. The chronicity of PE was not entirely clear in our patient. RV/LV diameter ratio > one indicated RV dysfunction and can be seen in right heart strain from acute PE or chronic sequelae of pulmonary hypertension [19]. A 2D echocardiogram also revealed high pulmonary artery pressure, which along with the chronology of pleural effusion, suggests a chronic element. Unfortunately, given the patient’s clinical instability later during his hospitalization, further diagnostic workup to clarify the chronicity of PE couldn’t be completed. Still, as mentioned above, it would be fair to state that the PE was acute on chronic in nature.

## Conclusions

In conclusion, our case highlights the importance of recognizing associations other than pneumonia in patients presenting with *E. coli* empyema and bacteremia, such as pulmonary thromboembolism and microaspiration, as seen in our patient. SBEM is rarely seen in patients without cirrhosis. Thus, a high index of suspicion should be maintained, especially when evaluating patients with unexplained pleural effusions, as it can prompt timely evaluation and management decisions.
